# Efficacy of intermittent hypoxic training on hemodynamic function and exercise performance in competitive swimmers

**DOI:** 10.20463/jenb.2018.0028

**Published:** 2018-12-31

**Authors:** Hun-Young Park, Won-Sang Jung, Jisu Kim, Hyejung Hwang, Kiwon Lim

**Affiliations:** 1 Physical Activity and Performance Institute , Konkuk University, Seoul Republic of Korea; 2 Department of physical education, Konkuk University, Seoul Republic of Korea

**Keywords:** Competitive swimmers, intermittent hypoxic training, hemodynamic function, exercise performance

## Abstract

**[Purpose]:**

Hemodynamic function is a parameter indicating oxygen delivery and utilization capacity and is an important determinant of exercise performance. The present study aimed to determine whether intermittent hypoxic training (IHT) ameliorates hemodynamic function and exercise performance in competitive swimmers.

**[Methods]:**

Twenty competitive swimmers (10 men, 10 women) volunteered to participate in the study. Participants were divided into the normoxic training (NT) group and the hypoxic training (HT) group and were subjected to training in a simulated altitude of 3000 m. We evaluated hemodynamic function profiles over 30 min of submaximal exercise on a bicycle and exercise performance before and after 6 weeks of training, which involved continuous exercise at 80% maximal heart rate (HR_max_) for 30 min and interval exercise at 90% of HR_max_ measured before training for 30 min (ten rounds comprising 2 min of exercise followed by 1 min of rest each round).

**[Results]:**

Significant changes in oxygen consumption (decrease) and end-diastolic volume (increase) were observed only in the HT group. Heart rate (HR), cardiac output (CO), and ejection fraction (EF) were significantly reduced in both groups, but the reduction rates of HR and CO were greater in the HT group than in the NT group. Exercise performance measures, including maximal oxygen consumption and 400-m time trial, were significantly increased only in the HT group.

**[Conclusion]:**

Our findings suggested that 6 weeks of IHT, which involved high-intensity continuous and interval exercise, can effectively improve exercise performance by enhancing hemodynamic function in competitive swimmers.

## INTRODUCTION

Since the Olympic Games were held at a high altitude in Mexico City in 1968, the usefulness of exercise training at altitude or under hypoxia for the enhancement of athletic performance has received considerable attention among athletes, coaches, and sports scientists^1,2^. Athletic performance is related to various parameters that can be altered by diversiform training methods at high altitude or under hypoxic conditions; these parameters include erythropoiesis, exercise economy, capillary density, hemodynamic function, and acid-base response in skeletal muscles^2,3^.

Altitude/hypoxic training is a common and popular practice among aerobic event athletes for improving exercise performance under normoxia. The most typical altitude/hypoxic training methods that have been proposed include the living high-training high (LHTH) and the living high-training low (LHTL) methods. Recently, intermittent hypoxic training (IHT) using artificial equipment has become an increasingly popular altitude/hypoxic practice; in this method, athletes live at or near sea level but undergo at training at 2,000 to 3,000 m to simulate hypobaric or normobaric hypoxic conditions^3-5^.

Among the various altitude/hypoxic training methods, the IHT may be of highest interest to athletes and coaches compared with the LHTH or the LHTL methods because the IHT method commonly involves shorter hypoxic exposure times, which typically last for < 3 h/day, two to five times per week, for 2 to 6 weeks^2,6^. In contrast to the LHTH or LHTL, the IHT does not provide a sufficient hypoxic stimulus to induce hematological changes. Therefore, the IHT does not improve exercise performance by increasing oxygen transportation capacity via erythropoiesis, which is characterized by increased red cell count and hemoglobin mass^7^. However, very interestingly, previous studies suggested that short-term exposure to hypoxic conditions combined with high-intensity exercise training improves athletic performance by enhancing the metabolic (i.e. blood lactate level, glycolytic enzyme and glucose transport, and acid-base regulation) and oxygen utilization capacity^2,4,7,8^. In addition, the IHT method can trigger various biochemical and structural changes in skeletal and cardiac muscles that favor the oxidative process and can enhance non-hematological parameters, such as exercise economy, acid-base balance, and metabolic response during submaximal exercise, ultimately leading to improved oxygen delivery and utilization capacity^2,4,7-10^.

However, the efficiency of the IHT for enhancing exercise capacity in various athletes under normoxia remains controversial. A few previous studies did not support the enhancing effect of the IHT on exercise performance^11-14^.These conflicting results could be attributed to several previous studies that did not verify the improvement in exercise performance based on the changes in hemodynamic function parameters, which serve as indicators of oxygen transport and utilization capacity^15,16^. In particular, most previous studies using IHT for swimmers focused on changes in exercise performance based on increased RBC count and Hb mass associated with erythropoiesis^13,17^.

Therefore, the present study aimed to investigate the effects of the IHT comprising warm-up, continuous exercise, interval exercise, and cool-down on hemodynamic function during submaximal exercise and exercise performance in competitive swimmers. We hypothesized that 6 weeks of hypoxic training is sufficient to elicit an increase in VO_2max_ and 400 m swimming performance by improving hemodynamic function profiles in competitive swimmers.

## METHODS

### Participants

A total of 20 moderately trained competitive swimmers (men = 10 and women = 10) who were registered in the Korea Swimming Federation and had an average of more than ten years of carrier were recruited as participants in the study (Table 1). The athletes did not participate in exercise and training under hypoxic conditions for 6 months prior to recruitment. The participants were non-smokers and did not have history of any musculoskeletal, cardiovascular, or pulmonary disease. Participants provided written informed consent after receiving sufficient explanation of the experiment and understanding the possible adverse effects prior to the start of the study. All procedures were conducted in accordance with the ethical standards of the responsible committee on human experimentation and with the Declaration of Helsinki. The study was approved by the Institutional Review Board of Konkuk University (HR-090) in Korea and was conducted according to the Declaration of Helsinki.

**Table 1.** Participant characteristics.

Variable	Before training	After training
Participant (N)		
NT group	10 (5 male, 5 female)	
HT group	10 (5 male, 5 female)	
Training condition (mm Hg)		
NT group	760 (sea level)	
HT group	526 (simulated 3000 m)	
Age (years)		
NT group	22.9 ± 3.9	
HT group	22.5 ± 2.6	
Height (cm)		
NT group	175.0 ± 9.9	
HT group	174.6 ± 9.2	
Weight (kg)		
NT group	69.2 ± 12.9	68.6 ± 12.9
HT group	72.7 ± 10.4	72.4 ± 11.0
Body fat (%)		
NT group	20.7 ± 4.0	20.5 ± 3.6
HT group	22.1 ± 4.3	21.9 ± 3.7

*Note*. NT = normoxic training, HT = hypoxic training

### Experimental design

**Figure 1.**
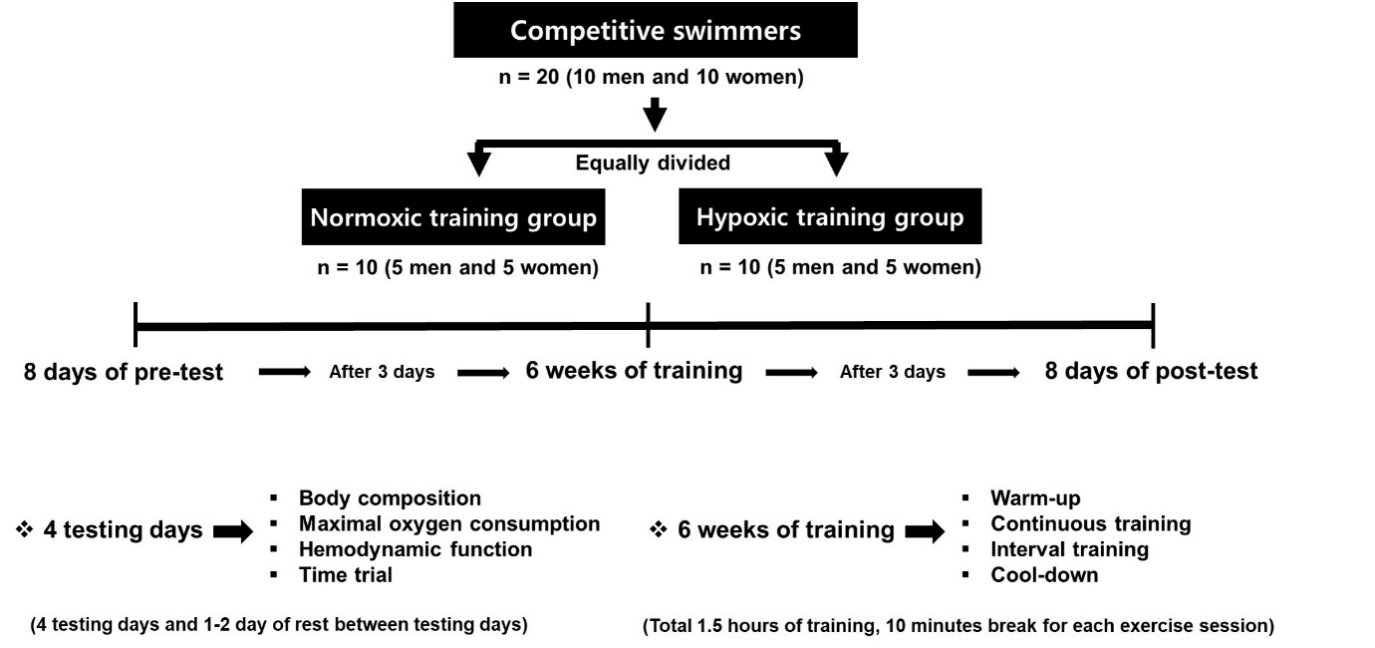
Experimental design of this study.

The study design was adapted from Park et al^2^. and is shown in Fig. 1. All competitive swimmers were equally divided by sex (five men and five women) and were assigned to one of two exercise training groups by body composition and exercise performance before training. The two treatment groups included a normoxic training group (performed exercise training at sea-level) and a hypoxic training group (performed exercise training at 526 mm Hg hypobaric hypoxic condition). Participants from the two treatment groups showed no significant differences in body composition and exercise performance Table 1. All testing procedures were performed in a 6.5 m (width) × 7.5 m (length) × 3 m (high) chamber with a temperature of 20 ± 2°C and humidity of 60 ± 2% regulated by a thermo-hygrostat (Submersible Systems, Huntington Beach, CA).

The present study was conducted based on the following schedule: 8 days of pre-test sessions (i.e., 3 test days and 1-2 days of rest between testing days), 6 weeks of training sessions under each environmental condition (i.e., normoxia or 526 mm Hg simulated hypoxic conditions; 3,000 m simulated altitude), and 8 days of post-test sessions. In the pre-test and post-test sessions, we measured body composition on the first test day between 8:00 and 9:00 am after at least 4 h of fasting. After approximately 2 h of meals and rest, maximal oxygen consumption (VO_2max_) and maximal heart rate (HR_max_) were measured using graded exercise testing following the BRUCE protocol on a treadmill under normoxic conditions. On the second test day, all participants underwent measurement of the exercise load (watts) corresponding to 75% and 90% of HR_max_ using graded exercise testing by the McArdle protocol on a bicycle under normoxic conditions. On the third day, hemodynamic function profiles of all swimmers were measured during 30 min of submaximal exercise on a bicycle under normoxic conditions. Exercise intensity was set at individual bicycle exercise load values (watts) corresponding to 75% of HR_max_ obtained at pre-test under normoxic conditions. On the fourth day, all swimmers underwent 400-m time trial in freestyle in an authorized indoor swimming pool in Seoul (50 m, length) at sea level.

In the training sessions, all swimmers performed the following four kinds of training in 90 min sessions: warm-up, continuous training, interval training, and cool down. The training frequency was 120 min per day for 3 days per week over a period of 6 weeks. Warm-up and cool down intervention were set at 50% of HR_max_ for each participant for the first 5 min and subsequently increased by 10% of HR_max_ every 5 min over a period of 15 min. The continuous training sessions comprised 30 min of continuous treadmill exercise corresponding to 80% of the HR_max_. The running velocity on a treadmill was changed using a heart rate monitor (Polar S610i, Finland) to match 80% of HR_max_. The interval training sessions comprised ten repetitions of interval cycling exercise (2 min of exercise corresponding to 90% of the HR_max_ and 1 min of rest). The measured bicycle load with 90% HR_max_ during maximal bicycle exercise at each environmental condition on the third pre-test day was set as the exercise intensity during the interval training session.

A study was designed to evaluate the effectiveness of hypoxic training vs. normoxic training on hemodynamic function and exercise performance. Therefore, before and after training, we evaluated hemodynamic function profiles (oxygen consumption; VO_2_, heart rate; HR, stroke volume; SV, end-diastolic volume; EDV, end-systolic volume; ESV, cardiac output; CO, and ejection fraction; EF) over 30 min of submaximal bicycle exercise corresponding to 75% of HR_max_ obtained before training. Exercise performance was evaluated before and after training by measuring VO_2max_ and performance in the 400-m time trial.

### Measurements

#### Height

Height was measured as the distance between the bottom of the foot and top of the head using an extensometer (PKS-1008, Japan).

#### Body composition

Body weight and % body fat were measured using an Inbody 770 instrument (Inbody, Korea). All participants fasted overnight prior to measurement of body composition. Participants wore lightweight clothing and were asked to remove any metal items.

#### Exercise performance

To evaluate exercise performance, VO_2max_ was measured pre- and post-intervention using the modified BRUCE protocol for graded exercise testing on a treadmill (S25T, STEX, Korea) equipped with a breath-by-breath auto metabolism analyzer (Quark CPET, Cosmed, Italy). The graded exercise test was initialized at 2.7 km∙hr^-1^ and 0% inclination and increased by 1.3 to 1.4 km∙hr-1 and 2% inclination every 3 min until voluntary exhaustion. All participants were considered to have reached their VO_2max_ if several of the following criteria were satisfied: a plateau or 'peaking over' in oxygen uptake, maximal heart rate was reached, respiratory exchange ratio of 1.15 or greater, and volitional exhaustion.

#### Hemodynamic function profiles during submaximal exercise

Hemodynamic function profiles were measured every min for 30 min of submaximal exercise on a bicycle. Aside from EF, the measurement value used was the summation value over a period of 30 min. EF measurements were conducted using the mean value at 30 min. VO^2^ was measured using an auto metabolism analyzer (Quark CPET, Cosmed, Italy), a breathing valve in the facemask form, and a bicycle (Monark Exercise AB, Vansbro, Sweden). HR, SV, EDV, ESV, CO, and EF were assessed non-invasively using a thoracic bioelectrical impedance device (PhysioFlow PF-05 Lab1, Manatec Biomedical, Paris, France), which was previously shown to provide reliable results in healthy men and chronic pulmonary disease patients^18^. The electrodes were positioned on the forehead, neck, xiphoid process, and lower ribs on the left side while avoiding the abdominal muscles, as these locations were suggested to be appropriate for human participants^1,15^.

### Statistical analysis

Means and standard deviations (SD) were calculated for each primary dependent variable. Normality of the distributions of all outcome variables were verified by conducting the Kolmogorov-Smirnov test. A two-way analysis (time × group) of variance with repeated measures of the “time” factor was used to analyze the effects of training programs on each dependent variable. If a significant interaction effect or main effect within time was detected, a Bonferroni post-hoc test was performed to identify within-group changes over time. Additionally, the paired t-test was used to separately compare post-intervention vs. pre-intervention values of dependent variables in each groups. P < 0.05 was considered statistically significant.

## RESULTS

### Hemodynamic function profiles

**Figure 2.**
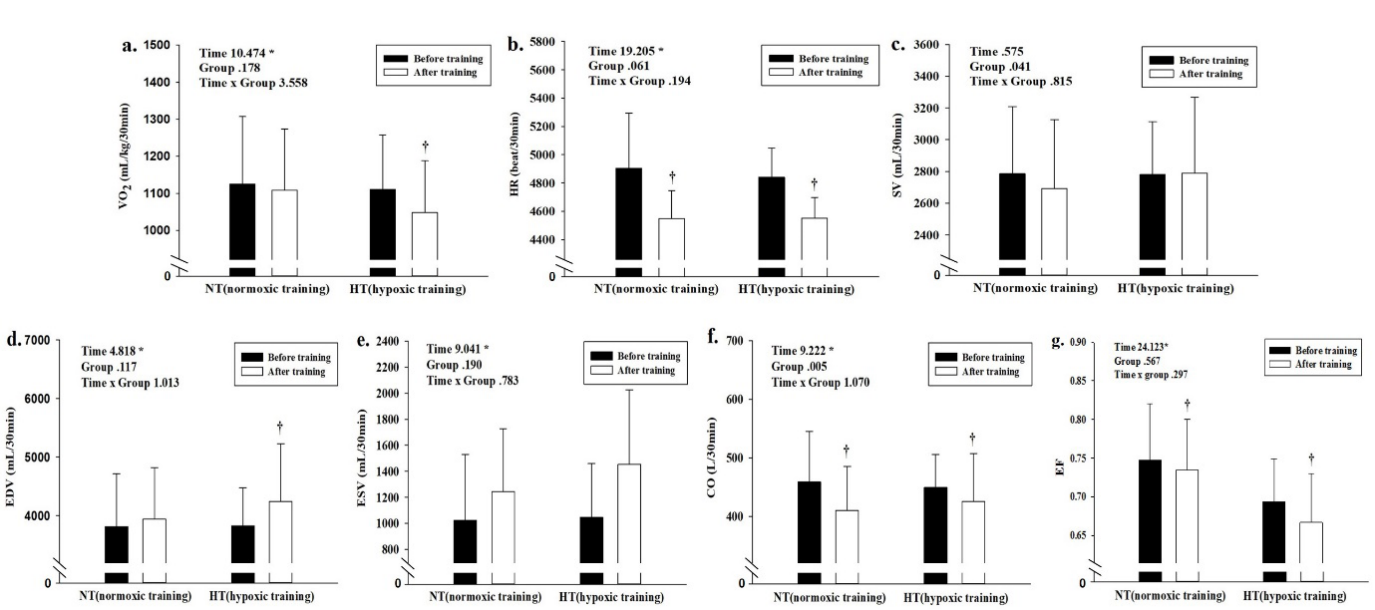
Changes in hemodynamic function during 30 min of submaximal exercise on a bicycle induced by training in the normoxic training (NT) and hypoxic training (HT) groups. a. oxygen consumption (VO_2_) b. heart rate (HR) c. stroke volume (SV) d. end-diastolic volume (EDV) e. end-systolic volume (ESV) f. cardiac output (CO) g. ejection fraction (EF). Bars indicate the mean ± SD. *: Significant (*p* < 0.05) interaction or main effect. †: Significant (*p* < 0.05) difference between before training and after training values in each treatment group.

Before and after intervention data for hemodynamic function profiles measured during 30 min of submaximal exercise on a bicycle are shown in Fig. 2. All hemodynamic function profiles showed no significant interactions; however, a significant main effect within time was observed in VO_2_ (F = 10.474, *p* < 0.005), HR (F = 19.205, *p* < 0.005), EDV (F = 4.818, *p* < 0.005), ESV (F = 9.041, *p *< 0.005), CO (F = 9.222, p < 0.005), and EF (F = 24.123, *p* < 0.005); the HT group showed a significant change (*p* < 0.005) in VO_2_ (decrease), HR (decrease), EDV (increase), CO (decrease), and EF (decrease) between before and after training. However, the NT group showed a significant decrease (*p* < 0.05) in HR, CO, and EF between before and after training. The reduction rates in HR and CO were greater in the HT group than in the NT group. In addition, post-hoc analysis showed no difference in ESV between the two treatment groups. We observed no significant interaction or main effect within time in SV.

### Exercise performance

**Figure 3.**
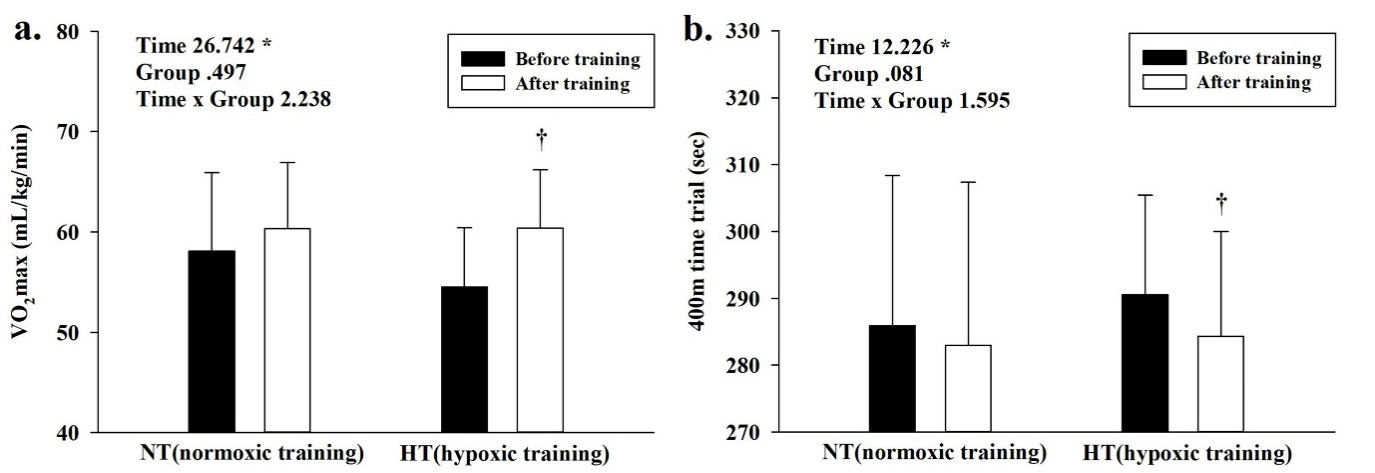
Changes in exercise performance (maximal oxygen consumption; VO_2max_ and 400-m time trial) induced by training in normoxic training (NT) and hypoxic training (HT) groups. a. VO_2max_ and b. 400-m time trial. Bars indicate mean ± SD. *: Significant (*p* < 0.05) interaction or main effect. †: Significant (*p* < 0.05) difference between the before training and after training values in each group.

Before and after training data for exercise performance in both groups are presented in Fig. 3. There was no significant interaction in exercise performance, but significant main effects within time were observed in VO_2max_ (F = 26.742, *p* < 0.005) and the 400-m time trial (F = 12.226, *p* < 0.005). Results of post-hoc analysis showed improved exercise performance in the HT group: VO_2max_ (*p* < 0.005) and performance in the 400-m time trial (*p *< 0.005) were higher only in the HT group.

## DISCUSSION

In general, the most typical altitude/hypoxic training methods [living high-training high (LHTH) or the living high-training low (LHTL)] improve athletic performance by enhancing oxygen delivery capacity, such as enhanced erythropoiesis (e.g. increased red cell volume and hemoglobin mass) by exposure to hypoxia for at least 16 h^6,18^. In recent years, the intermittent hypoxic training (IHT) using artificial equipment has become an increasingly popular altitude/hypoxic practice; in this method, athletes live at or near sea-level but train at 2,000 to 3,000 m simulated hypobaric or normobaric hypoxic conditions^3-5^. The IHT regime commonly involves shorter hypoxic exposure (approximately two to five sessions per week at < 3 h per session), lower cost, less effort, and shorter training time than the LHTH and the LHTL regimes^6^. 

Various studies examined the efficacy of the IHT on athletic performance and reported that the IHT does not improve blood delivery capacity by increasing the red cell volume and hemoglobin mass like the LHTH or the LHTL method^7^. However, the IHT effectively improves athletic performance by enhancing the metabolic (i.e. blood lactate level, glycolytic enzyme and glucose transport, acid-base regulation) and oxygen utilization capacity^2,4,7,8^. In addition, the IHT could induce various biochemical and structural changes in skeletal and cardiac muscles that favor oxidative process, which could in turn improve non-hematological parameters, such as exercise economy, acid-base balance, and metabolic response during submaximal exercise to ultimately improve oxygen delivery and utilization capacity^2,4,7-10^. However, the effect of IHT on athletic performance under normoxia remains an open question because of differences in measured dependent parameters and differences in the hypoxic training methods, such as the dose of hypoxic stimulus, type and intensity of training, participant training status, and timepoint of measurement of athletic performance following the IHT regime^14,19-22^. Therefore, based on the criteria proposed the IHT protocol, which was demonstrated to maximize exercise performance in the previous study^2,4,6,14^, our study investigated the efficacy of the IHT (< 3 h of hypoxic exposure for thrice a week over a period of 6 weeks) on improving hemodynamic function profiles and exercise performance in competitive swimmers. Particularly, multiple previous studies that investigated the IHT did not verify that IHT improved exercise performance based on the changes in hemodynamic function profiles, which indicate oxygen delivery and utilization capacity. Therefore, our study investigated the efficacy of the IHT (< 3 h of hypoxic exposure for thrice a week over a period of 6 weeks) with respect to hemodynamic function and exercise performance in competitive swimmers. Our current findings confirmed that the IHT regime, which comprised warm-up, continuous exercise, interval exercise, and cool down, improved exercise performance (e.g. VO_2max_ and 400-m time trial) in competitive swimmers by significantly enhancing hemodynamic function parameters (e.g. VO_2_, HR, EDV, and CO) during submaximal exercise.

Regarding hemodynamic function profiles, the significant decrease in VO_2_ during submaximal exercise indicated enhanced exercise economy. VO_2max_ and exercise economy (defined as the amount of energy per unit distance) are widely known as determinant factors of exercise performance^2,23-26^. Mechanisms that potentially contribute to enhanced exercise economy by various hypoxic training modalities include multiple activities that promote ATP re-synthesis (per 1 mole O2) and decrease in ATP levels at a given speed during exercise^2,12,23,27^. In addition, exercise training under hypoxic conditions leads to increased efficiency of oxygen utilization and higher energy availability, which in turn promote the invigoration of the parasympathetic nervous system via the activation of β-adrenergic receptors in cardiac muscles and improve EDV with enhanced venous return^23,28^. Our findings revealed that all training groups showed significantly lower HR and higher CO levels during submaximal exercise. However, EDV increased only by training in the HT group, and the positive effects of HR and CO were greater in the HT group than in the NT group. In addition, all treatment groups showed higher ESV. Although the NT and HT groups presented higher afterload (the pressure in the wall of the left ventricle during ejection), only the HT group showed higher preload (the pressure that stretches the right or left ventricle of the heart to maximum capacity under variable physiologic demands). In other words, greater ventricular relaxation dependent on EDV is caused by higher venous return and left ventricular volume through exercise training under hypoxia; these effects appear to be greater than exercise under normoxia. Therefore, our results suggested that a significant variation in VO_2_ (↓) and EDV (↑) via IHT improves VO_2max_ and 400 m time trial by increasing blood supply and oxygen utilization in skeletal and cardiac muscles. However, a significant improvement in SV as a result of increased EDV (Frank-Starling law) and a significant decrease in HR induced by the stimulation of the vagus nerve is not caused by hypoxic training compared with normoxic training. Although the hypoxic training modality is different, the current results are contradictory to those of a previous study that verified the improvement of the HR via the LHTL method^15,28^.

In summary, our findings confirmed that compared to NT, 6 weeks of IHT resulted in higher VO_2max_ and better performance in 400-m time trial by improving exercise economy and preload (a decrease in VO_2_ and an increase in EDV during submaximal exercise).

## CONCLUSION

Our results suggested that 6 weeks of the IHT (< 3 h of hypoxic exposure, thrice per week for 6 weeks), which comprised warm-up, continuous exercise, interval exercise, and cool-down for 90 min in simulated 3000 m hypoxic conditions (526 mmHg), is an effective training method for improving exercise performance (VO_2max_ and 400-m time trial) by enhancing hemodynamic function (VO_2_, HR, EDV, and CO) in competitive swimmers.
